# New Inventions

**Published:** 1896-09

**Authors:** 


### NEW INVENTIONS.

A horseshoe with mortised recesses for toe and heel calkings secured by lugs.

A soft-tread horseshoe, consisting of a shoe deeply grooved on the solar face next the inner border, to receive an elastic or yielding packing, with a similar pad between the heels, supported in rabbets formed in the upper face of the shoe.

A sectional horseshoe jointed at the toe, with a toe-calk fastened to each section.

A kicking-strap, secured in front to bit passing over the poll, then to the saddle through loops and through similar loops on each side fastened to the crupper, from whence they are carried to the shaft-bar and secured.

A horseshoe calk, wedge-shaped, fitted to a socket provided with a longitudinal channel to receive said calking; the socket provided with flanges to enable its welding to the ordinary shoe.

A wheel-tire, comprising an exterior casing of suitable flexible material and a compressed filling of felt.

A horseshoe calk-extractor.

A device for holding horses, consisting of a spiral lineholder-rod encircling the hub of a wheel.

A hame-fastener, consisting of a solid portion with loop extremity and a pointed section opening by a spring.

A harness-buckler and trace attachments.

A check-bit, consisting of straight mouthpieces ending in rings for adjusting straps, the mouthpieces having parallel extensions or side-bars connected in front by a curved piece, the mouthpiece of a continuous wire rod.

A harness-saddle, with adjustable back-pads pivoted thereto.

A detachable calk for horseshoes to prevent slipping, consisting of a toe-calk plate connected with projections from the rings of the shoe and regulated by a screw adjusted to said levers.

A collapsible top for vehicles.

A bottle-stopping device for sterilizing purposes, having a rubber cap, with a cap inside the neck, allowing the gas to escape during the sterilizing period.

A bottle-labelling machine.

A nose-bag, arranged to be held in position by sheaves at sides of bag fastened over the head and regulated by draw-strings carried backward and fastened to the collar.

An elastic-tread horseshoe, composed of recessed metal frame with apertures, a continuous rubber cushion in said recess connecting with a rubber cushion on the back through the apertures, this cushion resting between the metal frame and hoof.

A wagon-pole feedbox, made in halves, hinged together and secured by a depending clip.

A horseshoe, composed of two sections, a plate having slots for the reception of the lugs on the outer shoe, thereby forming a dovetail, and a pin or bolt serving to expand the outer shoe and lock the same.

A combined halter and blind, made from a piece of sheet-metal, and attached to the head-stall and sidestraps of the halter.

A vehicle-shaft, the front end of each ending in a terminal loop for the attachment thereto of the back-band, the loop being provided with a cross-stay for the attachment of the trace.

A harness-pad, having a backing of soft, absorbent material of felt and a lining of haircloth, the latter with its smooth side next to the animal's skin.

A horse-starting device, consisting of a screen adjusted with ropes or cables, carried to supports at opposite sides of the track, with a take-up device.

A trace-fastener, consisting of a body-portion with a jaw pivotally supported from the body, with a wire spring in jaw-portion.

A horseshoe with detachable calks, the calks having T-ends for securing to the shoes.

A hitching device, to be secured at one end to the horse, the other to a wheel, with a clamping lever device, with a stop adjustment to lock the wheel subsequently to stopping the horse.

A rubber-cushioned horseshoe, the rubber having long wires or fibres woven through it, the frame of the shoe having lugs to extend into the rubber and wire-netting, and the whole vulcanized.

An automatic feeding-box, with a hinged bottom, with a pivoted lever at one end and a roller-sustaining bottom at other end, with time gearing attachment and weight.

A suitably flanged, jointed, marsh horseshoe.

A breaking-rig, consisting of a surcingle with a fixed guide pendant therefrom, and a series of limiting-straps, with suitable attachments for securing to the fore and hind limbs.

A stand for shoeing horses.

